# Limitations of Flow Diverters in Posterior Communicating Artery Aneurysms

**DOI:** 10.3390/brainsci11030349

**Published:** 2021-03-09

**Authors:** Michelle F. M. ten Brinck, Luigi Rigante, Viktoria E. Shimanskaya, Ronald H. M. A. Bartels, Frederick J. A. Meijer, Ajay K. Wakhloo, Joost de Vries, Hieronymus D. Boogaarts

**Affiliations:** 1 Department of Neurosurgery, Radboud University Nijmegen Medical Center, 6500 HB Nijmegen, The Netherlands; Michelle.tenBrinck@radboudumc.nl (M.F.M.t.B.); luigirigante@gmail.com (L.R.); Vika.Shimanskaya@radboudumc.nl (V.E.S.); Ronald.Bartels@radboudumc.nl (R.H.M.A.B.); Joost.deVries@radboudumc.nl (J.d.V.); 2 Department of Radiology and Nuclear Medicine, Radboud University Medical Center, 6500 HB Nijmegen, The Netherlands; Anton.Meijer@radboudumc.nl; 3 Department of Neurointerventional Radiology, Tufts Medical Center, Boston, MA 02111, USA; ajay.wakhloo@lahey.org

**Keywords:** aneurysm, endovascular, flow diverter, posterior communicating artery

## Abstract

Background: Treatment of intracranial aneurysms with flow diverters (FDs) showed promising results. However, a subset of patients treated for posterior communicating artery (PComA) aneurysms has variable occlusion rates. Especially the fetal type-associated PComA aneurysms seemed to respond differently to treatment. We analyze our series of fetal type PComA aneurysms treated with a FD. The literature on this subject is reviewed. Methods: Data from patients treated with FD for all PComA aneurysms at the RadboudUMC Nijmegen were retrospectively analysed. Primary end-point was complete aneurysm occlusion at six months. Secondary end-points were clinical outcome, treatment safety, and results of secondary treatment after non-closure. The results for the fetal PComA aneurysms were compared to the literature. Results: Nineteen consecutive patients harboring 21 PComA aneurysms were treated. Three aneurysms had ipsilateral fetal type PCA (14.3%). Overall, none of the fetal type PcomA aneurysm showed complete occlusion versus 77.8% of the others (*p* = 0.03). Mortality and permanent morbidity rates were respectively 5.3% and 0%. Conclusions: FD treatment for PComA aneurysm with fetal type circulation seemed to be less effective compared to other types of PComA aneurysms. Flow characteristics at the PComA bifurcation are thought to be causative Alternative strategies should be considered as first line treatment.

## 1. Introduction

The use of flow diverting (FD) stents to treat intracranial aneurysms of the internal carotid artery has recently emerged, with promising results [1]. FDs reconstruct the lumen of parent vessels, serving as a frame on which endothelial cells can proliferate and induce thrombosis inside the aneurysm sac [2]. This technique has been proven to be effective in posterior communicating artery (PComA) aneurysms. However, a small subset of these aneurysms, the so called fetal-type PComA aneurysm does not seem to show occlusion and contrasting reports have been published. We analyzed our series of fetal type PComA aneurysms treated with a FD. The literature on this subject is reviewed.

## 2. Materials and Methods

All consecutive electivelly treated patients with the Surpass FD (Stryker Neurovascular, Fremont, CA, USA) for a PComA aneurysm between June 2010 and October 2015 (follow-up until 2020) at Radboud University Medical Center Nijmegen, were retrospectively analyzed. Procedures were performed by two senior hybrid neurosurgeons. PROCESS guidelines were followed for the collection and reporting of data. Patients harboring either unruptured PComA aneurysms or a previously ruptured PComA aneurysm, who had been acutely treated endovascularly (e.g., with coiling) without aneurysms full occlusion, were included. Review and approval of the study protocol was obtained from the local institutional review board (no. 2016-2419). Patient informed consent was not required, which was confirmed by the Radboudumc local institutional review board.

Type, location, size of the aneurysm, neck width, dome size, and aneurysm dome-to-neck ratio were measured and collected on the preoperative digital subtraction angiography (DSA). Presence of the PCA on the side of the aneurysm, diameter of the internal carotid, posterior communicating artery, and P1 segment, carotid-to-PComA ratio, as well as circle of Willis configuration according to Van Raamt were reported (normal configuration with PComA diameter <P1 diameter, transitional configuration with PComA diameter = P1 diameter, partial fetal type configuration with PComA diameter >P1 diameter, full fetal type configuration with ipsilateral P1 segment absent) [3]. The carotid diameter was calculated as the average between the diameter measured proximal and distal to the PComA aneurysm neck.

Patients received acetylsalicylic acid (100 mg daily) and clopidogrel (75 mg daily) 5 days prior to the procedure and maintained for 3 to 6 months after which lifelong acetylsalicylic acid only. During the procedure patients received a standard dosage low molecular weight heparin intravenously (3500–5000 IU depending on their weight). For more information regarding the Surpass FD we refer to other published literature [4].

Primary end-point was complete aneurysm occlusion at 6 months angiographic follow-up. In case of complete aneurysm occlusion at the first postoperative DSA, no further imaging was performed unless considered necessary. Occlusion rate was defined according to the Raymond-Roy (RR) grading scale [5]. Complete aneurysm neck coverage was checked and SFD proximal and distal wall apposition were evaluated. The results were assessed by 3 independent reviewers.

Secondary end-points were clinical outcome at 6-months follow-up, treatment safety and results of secondary treatment after initial non-closure. The following factors were recorded regarding treatment safety: primary adverse events reported included death, thromboembolic events, stroke and hemorrhage possibly related to the treatment. Secondary adverse events recorded were radiological complications (e.g., ischemic damage on imaging but no clinical symptoms), transient ischemic attack (TIA), new neurological deficits, and need for re-intervention for incomplete occlusion or device mispositioning. Retreatment strategy after non-closure was determined by the treating physicians.

Univariate analysis was performed to evaluate relation of PComA configuration, aneurysm dome-to-neck ratio, and size to aneurysm occlusion (SPSS version 22 software, SPSS Inc., Chicago, IL, USA). The other factors were judged as not relevant for aneurismal occlusion. Fisher’s exact text was used to analyse significance of rate differences between the fetal-type configuration group and others. The independent-samples *t*-test was used to analyse significance between mean differences in these groups. Data are represented by mean + standard deviation (range). Statistical significance was assumed if *p* < 0.05.

A literature search in Medline on FD treatment of fetal-type PComA aneurysms was performed ((((flow div* OR flowdiv* OR flow-div* OR pipeline OR PED OR silk OR surpass OR fred OR flow re-directing OR flow redirecting OR flow-redirection OR p64 OR derivo OR flow modulation OR tubridge)) AND posterior communicating)) AND fetal[Text Word]. Data were extracted on occlusion rate and follow-up time.

## 3. Results

### 3.1. Patients and Clinical and Radiological Outcomes

Nineteen consecutive patients, two of whom harboring bilateral PComA aneurysms (21 aneurysms total), were electively treated with Surpass FD. Fifteen patients were female and four male; mean age of 56.4 (SD + 9.9) years (Table 1). One procedure had to be aborted due to thromboembolic events prior to FD deployment, but this patient had a successful procedure 3 months later (22 procedures total). For other demographics of these patients we refer to Table 1. Three aneurysms (14.3%) had an full fetal type circulation and 1 (4.8%) had a transitional configuration, the remainders (17; 81.0%) had a normal configuration.

FD deployment was successful in all cases except one (95.5%), in which the procedure was aborted due to thromboembolic events, as mentioned previously. Two cases included additional coiling within the same procedure as FD placement (9.1%): One concerned the patient with a transitional configuration and giant PComA aneurysm. This patient’s procedure consisted of partial aneurysm coiling prior to delivery of the flow diverter all within the same procedure. The other patient was treated with additional aneurysm coiling within the same procedure after FD placement due to retrograde filling of the aneurysm after FD deployment.

Mean total clinical and radiologic follow-up were 17.3 + 15.5 months (4–69) and 19.1 + 18.0 months (4–69), respectively. (Table 1) Six months imaging follow-up results were available for all patients.

Mortality rate was 0% and no delayed aneurysm rupture occurred at follow-up. Procedural complications occurred in 1/22 procedures (4.5%), however this did not result in any permanent deficit. Of the five patients receiving secondary treatment one experienced a procedural complication: Intraoperative DSA showed a perforation of a small M2 branch which resolved spontaneously. There was no clinical sequelae. The total procedural complication rate was therefore 7.4% (2/27).

Periprocedural or late complications were experienced by four patients, however this only led to permanent deficit in one patient (4.5%). (Table 1). One patient experienced amaurosis fugax several days after clopidogrel discontinuation 6 months after the procedure. The dual antiplatelet regimen was restarted indefinitely, there were no persisting deficits. mRS at 6 months was <3 for all patients. There were no changes in clinical condition after six months with mean follow up time of 17.3 months.

At 6 months follow-up, full aneurysm occlusion (Raymond-Roy 1) was observed in 77.8% of the patients with a non-fetal configuration (14/18). For the other four, aneurysm occlusion was graded as RR 2 for two aneurysms and RR 3 for two aneurysms.

### 3.2. Fetal Type PcomA Patients

**Figure 1:**
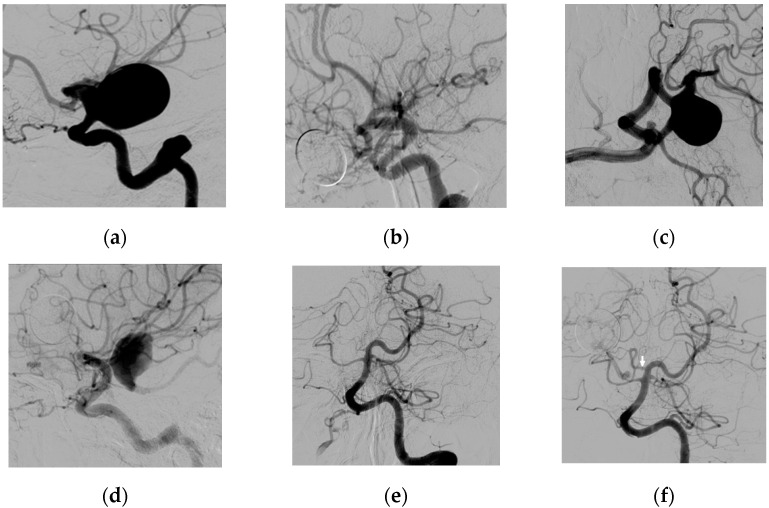
Images of a middle-aged patient with bilateral PComA aneurysms and full fetal type PCA configuration on the right side: (**a**) Lateral angiographic left ICA injection before treatment; (**b**) Six months follow-up DSA showing complete occlusion of the left PComA aneurysm; (**c**) Lateral angiographic right ICA injection before treatment; (**d**) Six months follow-up DSA showing treatment failure of the right PComA; (**e**) Left vertebral artery AP injection showing full fetal type configuration on the right side; (**f**) Follow-up DSA made 1.5 year after initial FD placement on the right with a now visible P1 segment (indicated by the arrow).

Occlusion in patients with fetal type configuration did not occur (0%; 0/3, all RR3). Two of these patients showed a shift in hemodynamics during follow-up: For one patient, initially, the P1 segment was not visible on DSA, however a DSA made at 1.5 years follow-up showed an open P1 and persistent filling. Maximum aneurysm size at that moment was 16 mm. Coiling via the posterior circulation was advised to the patient, however, the patient refused coiling and wanted to be treated with nothing but another flow diverter which eventually was done. This initially led to a significant decrease in size (6 mm) at 2 months follow-up, however at 7 months (total 25 months) follow-up maximum it was increased back to 8 mm (Figure 1).

For the second patient coiling was not an option due to absence of a sufficient caliber of the P1 segment to occlude the PComA, therefore a second flow diverter was placed. This secondary treatment had no influence on aneurysmal occlusion and no hemodynamic shift occurred after 30 months follow-up (no filling of the PCA via the P1).

The aneurysm of the third patient showed substantial decrease in size after FD placement, however the occlusion grade was still Raymond-Roy 3 after 25 months follow-up. MRA at 60 months follow-up showed a residual aneurysm with a maximum size of 16 mm and a now open P1 segment.

### 3.3. Analysis

No multivariate analysis was performed due to the low number of aneurysms (*n* = 21). PComA size, fetal configuration, and presence of P1 segment were interrelated. Therefore, only PComA configuration, dome-to-neck ratio and aneurysm size were selected as the most relevant variables. Difference in occlusion (RR) between non-fetal or intermediate type and full fetal type PCA associated PComA aneurysms was statistically significant (Fisher’s exact test, *p* = 0.03). Dome-to-neck ratio was not significantly related to aneurysm occlusion (Kruskal Wallis test, *p* = 0.76). There was no trend for non-occlusion in aneurysms with a size of >10 mm versus <10 mm.

### 3.4. Literature

Literature search revealed six studies on flow diversion for PComA aneurysms with a fetal type PCA [6,7,8,9,10,11,12]. In total 51 patients including the present series have been reported. Complete occlusion is accomplished in 16 (31.4%). Follow up is given in all but one, and from 5.0 to 27.6 months (Table 2).

## 4. Discussion

Our study presents a case series of PComA aneurysms of patients treated with the Surpass FD with a special focus on fetal type configuration. The prevalence of this anomaly is 4–26% [3]. Occlusion was not accomplished in the fetal type associated PcomA aneurysms by using flow diversion technique. PComA aneurysms of patients with a non-fetal configuration can be treated effectively with flow diversion [14]. Our literature search showed that reported results of all studies on flow diversion for fetal type associated PComA aneurysms are disappointing with a combined occlusion rate of 31.4% [4,6,7,8,9,10,11,12,15,16]. However, the two most recently published studies report rates of 75% and 43.8%, respectively [11,12].

Alternative strategies for fetal type PcomA aneurysms might have drawbacks. Standard coiling is generally not advised for large aneurysms due to the high recurrence rates. A series of nine fetal type PCA associated PComA aneurysms (7 with size <10 mm) treated with several endovascular techniques (no FDs) showed near complete or complete occlusion in eight patients without occurrence of procedure-related morbidity or mortality [17]. However in our series the size of the aneurysm was larger than 10 mm, which makes them less suitable for bare coiling.

Although clipping has shown high occlusion rates, a higher procedure-related morbidity has been reported compared to endovascular treatment in general [15,18]. A study including 30 fetal type associated PComA aneurysms of which 24 treated with clipping and 6 endovascularly by coiling reported a hemorrhage and/or stroke rate of 33.3% (8/24) for clipping versus 16.7% (1/6) for endovascular treatment. Full occlusion rates were respectively 91.7% and 83%. The aneurysms in this series were relatively small (mean aneurysm size 7 mm). Neck-to-dome ratios were not given [19]. Furthermore clipping may not always be feasible due to aneurysm configuration.

Possible explanations for the low occlusion rate of fetal type associated PComA aneurysms treated with flow diversion would be the continuous blood “sumping” from the ICA to the large caliber PComA across the FD and the backflow from the PCA territory through the PComA, resulting in diminished flow-diverting effect into the aneurysm sac [6,16].

The use of flow diverters for full fetal type PComA aneurysms should be restricted. Several alternative strategies should be considered like clipping, wrapping or stent assisted coiling [20]. In special cases, for example in complex or very large aneurysms with high surgical risks or because of high recanalization rates for endovascular coiling, an alternative strategy including FD can be considered as an option. Placement of a FD can induce a hemodynamic shift resulting in an opening of the P1 segment. Once sufficient in size, coiling via the posterior circulation and occlusion of the PComA origin at the carotid artery can be performed. An additional FD as second procedure to increase demand through the hypoplastic p1 segment can be considered. In cases were these strategies cannot be performed other additional surgical strategies should be considered like trapping and auxiliary revascularization [21].

Limitations of this study include the relatively small number of patients and its retrospective nature with all the corresponding biases that come with this study design. The results might be related to the implant used. However, in all other series reporting non-closure another device was used.

## 5. Conclusions

Fetal type circulation is negatively correlated with aneurysm occlusion by flow diverter treatment for PComA aneurysms. Elective microsurgical clipping or alternative or adjunctive endovascular strategies should be considered as first line treatment. Future research is needed to select subgroups who can benefit most from FD treatment.

## Figures and Tables

**Table 1:** Baseline characteristics and results.

		PCA Configuration		All Patients	*p*-Value ^1^
		Normal and Transitional Configuration	Fetal Configuration		
Number of patients/aneurysms		17/18	3/3	19 ^a^/21	NA
Mean age in years (SD, range)		55.9 (9.8, 40–80)	59.3 (12.3, 49–73)	56.4 (9.9, 40–80)	0.59 *
Female sex (*n*)		14 (77.8%)	2 (66.7%)	15 ^a^ (78.9%)	1.00
Size	Small (<10 mm)	8 (44.4%)	0 (0%)	8 (38.1%)	0.26
	Intermediate (10–20 mm)	7 (38.9%)	1 (33.3%)	8 (38.1%)	1.00
	Large (≥20 mm)	3 (16.7%)	2 (66.7%)	5 (23.8%)	0.13
Mean neck size in mm (SD, range)		3.5 (1.2, 1.7–6.3)	7.7 (5.9, 4.0–14.6)	4.1 (2.6, 1.7–14.6)	0.34 *
Mean dome-to-neck ratio in mm (SD, range)		3.4 (1.9, 1.4–9.3)	4.3 (4.4, 1.6–9.4)	3.5 (2.2, 1.4–9.4)	0.76 *
Number of wide-neck aneurysms (Neck ≥ 4.0 mm or dome-to-neck ratio < 2.0)		9 (50.0%)	3 (100%)	12 (57.1%)	0.23
mRS at 6 months ≤ 2		18 (100%)	3 (100%)	21 (100%) ^b^	NA
Initial closure at 6 months (%)		14 (77.8%)	0 (0%)	14 (66.7%)	0.03
Retreatment (*n*/*n* aneurysms not occluded at 6 months, (%))		3/4 (75%)	2/3 (66.7%)	5/7 (71.4%) ^c^	1.00
Full aneurysm occlusion at last follow-up (%) (Mean follow-up ± SD, range (months))		17 (94.4%)	0 (0%)	17 (81.0%) 19.1 ± 18.0 (4–69)	0.00
Patients with complications	Total	4 (23.5%)	0	4 ^d^/19 (21.1%)	1.00
	Procedural	1 (5.9%)	0 (0%)	1/19 (5.3%)	
	Periprocedural (≤30 days)	3 (17.6%)	0 (0%)	3/19 (15.8%)	
	Late (>30 days)	1 (5.9%)	0 (0%)	1/19 (5.3%)	

^1^ For the marked values (*) the independent samples T-test was used. For the other cases the Fisher’s exact test was used and the 2-sided *p*-value is presented. Significant *p*-values are marked bold. ^a:^ 1 patient with bilateral PComA aneurysms had a fetal type circulation on the right side and normal type circulation on the left side, therefore this patient is represented twice. ^b:^ Since 2 patients were harboring bilateral PComA aneurysms and these were treated on different moments, both these patients had two moments of 6 months follow-up, thus adding up to 21 instead of 19. ^c:^ Of the patients with non-fetal type configuration: For 1 patient with a small neck remnant an expectative policy was agreed on. Last follow-up still showed an unchanged small neck remnant. Two patients were additionally coiled via the posterior circulation (1 with neck remnant at 37 months after initial treatment and 1 with residual aneurysm at 14 months after initial treatment) leading to full occlusion in both. One patient with a residual aneurysm received a 2nd FD since a DSA made 21 months after initial treatment showed distal foreshortening of the FD (not seen on previous imaging). Consequently the aneurysm neck was not fully covered anymore. 6 months after placement of 2nd FD the aneurysm was fully occluded. ^d:^ Of which leading to permanent morbidity in 1 patient and transient (all resolved <24 h) morbidity in 3. 1 patient had both a procedural and periprocedural complication, so overall, the five complications occurred in four patients. FD = Flow diverter; mRS = modified Rankin Scale; PCA = Posterior cerebral artery; PComA = Posterior communicating artery; SD = Standard deviation.

**Table 2:** Literature on flow diverter treatment for fetal type PCA associated PcomA aneurysms.

Study, Year [Reference]	Number of Cases	FD Type	Aneurysm Size (Median) in mm ± SD (Range)	Aneurysms with Complete Occlusion (%)	Follow-Up Time in Months (Mean)
Tsang et al., 2015 [6]	4	PED	7.6 ± 3.3 (4.4–11.0) *	0 (0%)	NR
Zanaty et al., 2016 [7]	3	PED	12.0 ± 2.0 (10.0–14.0) *	0 (0%)	5.0
Daou et al., 2017 [8]	6	PED	NR	4 (66.7%)	6.0
Roy et al., 2017 [9]	9	PED	7.6 ± 3.8 (4.0–15.0)	1 (11.1%)	11.7
Wallace et al., 2017 [10]	6	PED	7.7 ± 4.5 (5.4–16.0)	1 (16.7%) ^a^	20.0
Enriquez-Marulanda et al., 2019 [11]	4	PED	NR	3 (75.0%)	11.9
Rinaldo et al., 2019 [12] ^b^	16	NR	12.1 ± 8.7 (4.0–35.0) ^c^	7 (43.8%)	22.8 ^d^
Present series	3	Surpass	26.0 ± 17.0 (10.0–44.0)	0 (0%)	27.6
TOTAL	51	N.A.	N.A.	16 (31.4%) ^a^	N.A.

PED: Pipeline Embolization Device; N.A.: Not applicable; NR: Not reported. *: Data for 1 aneurysm missing. ^a^: In the study of Wallace et al. another aneurysm was retreated after 6 months with a second PED and occlusion was demonstrated 36 months after the second treatment, yielding an overall occlusion rate of 33.3% (2/6) and for all studies joint: 33.3% (17/51) [10]. ^b^: The study of Kan et al. [13] Is incorporated in this study population. ^c^: Mean size. ^d^: Total follow-up time in patients months. In this study median time to occlusion for patients with and without a fetal configuration was 51 and 6 months, respectively.

## Data Availability

The data presented in this study are available on request from the corresponding author.
